# Developing trauma resilient communities through community capacity-building

**DOI:** 10.1186/s12889-021-11723-7

**Published:** 2021-09-16

**Authors:** Todd P. Gilmer, Kimberly Center, Danielle Casteel, Kyle Choi, Debbie Innes-Gomberg, Amy E. Lansing

**Affiliations:** 1 Herbert Wertheim School of Public Health and Human Longevity Science, University of California, San Diego, La Jolla, CA USA; 2 Los Angeles County Department of Mental Health, Los Angeles, CA USA; 3 Department of Psychiatry, University of California, San Diego, La Jolla, CA USA; 4 Department of Sociology, San Diego State University, San Diego, CA USA

## Abstract

**Background:**

Trauma is a significant public health issue, negatively impacting a range of health outcomes. Providers and administrators in public mental health systems recognize the widespread experience of trauma, as well as their limited ability to address trauma within their communities. In response, the Los Angeles County Department of Mental Health funded nine regionally based community partnerships to build capacity to address trauma. We describe partnership and community capacity-building efforts and examine community impact, defined as successful linkages to resources and changes in stress tolerance capacities among community members.

**Methods:**

We conceptualized community capacity-building as dissemination of trauma-informed education and training, community outreach and engagement, and linkage of community members to resources. We measured trauma-informed trainings among partnership members (*N* = 332) using the Trauma-Informed Organizational Toolkit. Outreach, engagement and linkages were documented using Event and Linkage Trackers. We examined changes in the type of successful linkage after the issuance of statewide mandatory restrictions in response to COVID-19. We examined changes in stress tolerance capacities among community members (*N* = 699) who were engaged in ongoing partnership activities using the 10-item Conner-Davidson Resilience Scale; the 28-item Coping Orientation to Problems; and the pictorial Inclusion of Community in Self Scale.

**Results:**

Training and education opportunities were widespread: 66% of members reported opportunities for training in 13 or more trauma-informed practices. Partnerships conducted over 7800 community capacity-building events with over 250,000 attendees. Nearly 14,000 successful linkages were made for a wide range of resources, with consistent linkage success prior to (85%) and during (87%) the pandemic. In response to COVID-19, linkage type significantly shifted from basic services and health care to food distribution (*p* < .01). Small but significant improvements occurred in coping through emotional and instrumental support; and sense of community connectedness (*p* < .05 each).

**Conclusions:**

Community-based partnerships demonstrated effective capacity-building strategies. Despite the pandemic, community members did not report reduced stress tolerance, instead demonstrating gains in external help-seeking (use of emotional and instrumental supports) and perception of community connectedness. Future work will use qualitative methods to examine the impact of community capacity-building and the sustainability of this approach for addressing the impact of trauma within communities.

## Background

Trauma is a significant public health issue, negatively impacting a range of health outcomes that disproportionately impact vulnerable populations including individuals and families with low incomes, those who come from racial, ethnic, immigrant or sexual minority backgrounds, and those who are homeless [1]. Consistent with other countries, a large percentage of the United States (US) population has experienced significant adversity, including stressors such as life-threating trauma (interpersonal violence, natural disasters), potentially traumatic events (emotional abuse, non-violent loss), or exposure to poverty, historical trauma, systemic racism, discrimination (racial, gender-identity, sexual orientation) or disenfranchisement (lack of opportunities, power or political representation) [2–4]. Many potential traumas have both emotional and physical consequences (assault, car accidents).

The development of symptoms due to adversity exposure varies by the type and severity of the event, cumulative or repeated exposure, individual-level characteristics (age at exposure, access to individual, social and community resources or community strain), and intergenerational transmission [5, 6]. Symptoms and functional impairment (maladaptive coping strategies, decreased school or work performance, relationship dysfunction) are often worse in response to cumulative adversity exposure and poly-victimization [7–9]. Trauma amplifies health disparities, taxing already scarce or strained individual, social and community resources [10, 11]. The ongoing COVID-19 pandemic, itself a life-threatening event, has further drained resources, increasing the number of individuals, families and communities experiencing unemployment, lost health insurance, and food insecurity [12]. Although the COVID-19 pandemic affects society globally, its impact is especially severe in more vulnerable populations and communities [13].

Mental health providers and administrators both recognize the widespread experience of trauma in their communities, as well as their limited ability to address community-wide trauma within traditional community mental health centers [14]. Exposure to trauma simultaneously increases the level of a community’s needs while reducing trust in public health and social services [15]. In recent years, public mental health agencies have increased their focus on trauma-informed care: an approach to delivering mental health services that is sensitive to the social, psychological and biological consequences of trauma, with an increased eye towards cultural sensitivity and humility [16]. Trauma-informed principles and practices reduce the impact of trauma through culturally competent trust-building, safety, transparency, empowerment and collaboration which promote resilience, coping, and social connectedness [17]. A trauma-informed lens recognizes the importance of community level and contextual factors which affect both exposure to, and recovery from, a range of adverse and traumatic events.

In response to these realities, the Los Angeles County Department of Mental Health (LACDMH) funded nine regionally based community partnerships to build capacity to address trauma within their communities. Community capacity-building identifies and strengthens existing assets and skill sets within a community, establishing new collaborations to address emerging issues, and leveraging existing resources in a sustainable manner [18]. These community-embedded partnerships are implementing one or more of seven proposed primary trauma-informed community capacity-building strategies that focus on specific populations such as parents of young children, transition age youth, geriatric populations, and multigenerational families. Each strategy involves community outreach, engagement, and linkages to resources among community members, while addressing issues such as healthy parenting skills, youth homelessness, or trauma-informed professional development for public school educators. Collectively, the strategies were designed to facilitate capacity-building goals by addressing the consequences of trauma across the lifespan and increasing awareness of, and access to, resources and supports for vulnerable and underserved groups by communities rather than governmental institutions.

This study is part of a larger, longitudinal, mixed methods evaluation of the trauma-informed capacity-building initiative. The aim of the present study is to use quantitative data collected during the initiative to 1) quantify trauma-informed capacity-building efforts by partnerships and 2) examine the community impact of these capacity-building efforts in the most populous county in the US. Future qualitative work from this initiative will employ interviews and focus groups with partnership and community members to further examine the implementation of trauma-informed capacity-building, its impact on the community, and the sustainability of the initiative.

## Methods

This paper employs quantitative data from January 2018 through October 2020 to evaluate capacity-building to address trauma in communities and the subsequent community impact. Partnership capacity-building was captured through partnership meetings and trauma-informed trainings for partnership members, while community capacity-building was captured through community outreach, engagement and trainings reflecting the implementation of strategies focused on community needs. Community impact was assessed by successful linkages among community members and changes in stress tolerance capacities that support trauma resiliency, coping and connectedness among a subset of community members who were engaged in ongoing partnership activities.

### Study sample

Participants are captured at two levels in our quantitative evaluation: partnership members and community members. As of August 2020, the nine regionally based community partnerships included 531 unique partnership members from 100 community-based organizations. These organizations represented a variety of missions including city agencies (*N* = 10), education (*N* = 6), health care (*N* = 7), housing (*N* = 15), leadership training (*N* = 2), legal services (N = 2), mental health care (*N* = 9), ministry (*N* = 4), wellness (N = 2), and social services, both general (*N* = 13) and focused more specifically on children (*N* = 12), youth (*N* = 7), and families (*N* = 11). Among partnership members, 332 (63%) provided data on training in trauma-informed practice.

Community members were represented across the nine regions of Los Angeles County. As of October 2020, partnership sponsored capacity-building activities reached over 250,000 community members; this number is not unduplicated since identifying information was not collected from community members engaging in these activities. Nearly 14,000 linkages to services were made for 3609 unique community members. A total of 859 unique community members were engaged in ongoing partnership activities; 669 (78%) completed at least two assessments for three measures of stress tolerance capacity within six months of participation.

### Training in trauma-informed practice

Training in trauma-informed practices is core to capacity-building designed to offset the impact of trauma [19]. Trauma-informed practices arise from a strengths-based framework that emphasizes that all system responses should be grounded in a fundamental understanding of the impact of traumatic events on brain development, cognitive capacities, emotions, behaviors, and health outcomes. Trauma-informed practices foster resiliency and incorporate the Substance Abuse and Mental Health Services Administration’s (SAMHSA) ‘4 Rs’: Realizing the impact of trauma, Recognizing the signs of trauma, developing Trauma-Responsive systems, and Resisting re-traumatization [16].

We measured training in trauma-informed practices among partnership members using the Trauma-Informed Organizational Toolkit (TIOT). The TIOT was the result of a collaborative effort by the Center for Mental Health Services, National Child Traumatic Stress Network, W.K. Kellogg Foundation, Daniels Fund and SAMHSA to develop a tool that organizations can use to examine their current practices and take specific steps to become more trauma-informed [20]. The TIOT assesses organizational opportunities for education and training in trauma-informed principles and practices. Partnership members were asked to consider whether their partnerships incorporate each of 19 content areas related to trauma-informed best practices, using a Likert scale to indicate their opportunity to receive training on each topic. We considered responses of “agree” or “strongly agree” to indicate that an individual had education or training opportunities on that topic. The TIOT was tailored by removing one question about intake assessments (which are not used in this evaluation) and adding three questions to address educational components specific to the initiative: two questions on the relationship between poverty and trauma and one question on the use of a common language to describe the impact of trauma. The TIOT was administered in August 2019, February 2020, and August 2020. We used each member’s most recent assessment to provide descriptive statistics on the extent of trauma-informed training within the partnership. We used generalized estimating equations to examine changes in training among the partnerships over time [21, 22].

### Capacity-building activities and linkage to services

The process of outreach, engagement, and linkages to services was documented by partnership staff using registry-based tracking systems. An Event Tracker collected the name, date, and type of each event, and the number of people who attended. We used these data to quantify the number of capacity-building events and the numbers of participating community members. Community members participating in community events may receive referrals to specific resources or services. Partnership staff followed up on referrals to determine if the individuals were successfully linked with needed services and support. The Referral and Linkage Tracker documented referrals made for specific individuals and tracked whether the linkage was successful, unsuccessful, or in progress. We used data from the Referral and Linkage Tracker to quantify the number and types of successful linkages. We compared the distribution of linkages by type, prior to and post implementation of statewide mandatory restrictions in response to COVID-19, which were issued on March 20, 2020, using a chi-square test.

### Resilience, coping skills, and social connectedness

Some community members who participate in outreach engagement events later engage in ongoing activities or programs. Partnership staff aim to develop relationships with these individuals to learn more about their individual or family needs and strengths, and to build their skills to support resilience to trauma and stressful events, coping, and social connectedness. We used three established measures of stress tolerance capacities to evaluate potential changes in trauma-related resilience, coping skills, and social connectedness.

The 10-item Conner Davidson Resilience Scale (CD-RISC 10) is a unidimensional scale specifically capturing stress- and trauma-related resiliency using a 0 (“not at all true”) to 4 (“true nearly all the time”) Likert scale, with higher scores reflecting stronger resiliency (range: 0–40). The 28-item Coping Orientation to Problems (Brief COPE) assesses how individuals respond when they confront challenging and stressful life events. The 28 items reflect 14 two-item scales using a 1 (“I usually don’t do this at all”) to 4 (“I usually do this a lot”) Likert scale, with scale scores ranging from 2 to 8. We focused on scales reflecting external help-seeking (Use of Emotional Support and Informational Support) and internal empowerment (Active Coping, Planning), with higher scores reflecting greater coping strength. The 1-item pictorial Inclusion of Community in Self Scale (ICS) has been found to differentially measure community connectedness, separate from close/intimate relationships, with a 1 (separate circles) to 6 (completely overlapping circles) visual scale [23–25].

Self-reports were available in English and Spanish. We compared changes in these scales among community participants with two or more responses, between 30 and 180 days apart (*N* = 699), using paired t-tests. Only 12% (*N* = 74) of community member participants completed both their baseline and follow-up self-reports prior to the stay-at-home orders issued in response to COVID-19; 28% (*N* = 164) completed baselines prior to COVID-19 with follow-ups during COVID-19 and 60% (*N* = 359) completed all measures during COVID-19. Initially, self-reports were completed in-person (hard copies, electronically). During COVID-19, administration was more varied (remote interviews, computerized surveys).

## Results

Table 1 lists the capacity-building strategies used by the nine partnerships as their framework to develop trauma resilient communities. Most partnerships focused on children and youth: five partnerships employed strategy 1 aimed at children ages 0–5, four partnerships employed strategy 2 aimed at elementary school age children, and five partnerships employed strategy 3 aimed at transitional age youth ages 16–25. One partnership each employed strategies 4–6 and two partnerships employed strategy 7. Most partnerships addressed multiple strategies: one partnership employed four strategies, seven partnerships employed two strategies, and one partnership employed a single strategy.

**Table 1:** Community Capacity-Building Strategies used to Develop Trauma Resilient Communities among Nine Regionally Based Partnerships in Los Angeles County

Strategy 1: Building Trauma-Resilient Families employs outreach and engagement to caregivers with young children ages 0–5 who have experienced trauma and/or are at risk for trauma. Activities focus on enhancing the caregiver-child relationship, creating a connection with the community, and increasing knowledge about the impact of trauma on development and available community resources and services.	
Strategy 2: Trauma-Informed Psychoeducation and Support for School Communities focuses on developing training curriculums for Early Care/Education (EC/E) staff and school personnel to increase knowledge of behaviors and symptoms of stress and trauma, as well as trauma-informed coping techniques.	
Strategy 3: Transition Age Youth (TAY) Support Network employs youth peers to conduct outreach and engagement other youth ages 16–25 years old who may be socially disconnected and at risk of and/or experiencing mental illness or homelessness related to trauma.	
Strategy 4: Coordinated Employment within a Community is developing a network of community businesses that will offer job opportunities and skills training for youth, adults, and older adults with employment goals who have experienced trauma and/or are at risk for trauma or homelessness.	
Strategy 5: Community Integration for Individuals with a Mental Illness with Recent Incarcerations or Who Were Diverted from the Justice System provides community supports to facilitate community reintegration for youth, adults, and older adults who have experienced trauma or mental illness and histories of incarcerations or juvenile justice system.	
Strategy 6: Geriatric Empowerment Model (GEM) establishes a Senior Empowerment Center, which provides supportive services and education for older adults (60+) who are currently experiencing homelessness and/or trauma from being homeless.	
Strategy 7: Culturally Competent Activities for Multigenerational Families Experiencing Trauma addresses community or societally induced intergenerational trauma through culturally appropriate outreach, education and engagement, and non-traditional culturally relevant family healing activities.	

Table 2 summarizes training in trauma-informed practices among 332 partnership members (63%) who completed the TIOT. Opportunities for trauma-informed education and training was widespread: 28% of members reported that their partnership offered training on all 19 trauma-informed practices, and an additional 38% reported training opportunities on 13–18 practices. Most partners reported that their partnership offered training to impart foundational knowledge related to understanding traumatic stress (85%), how traumatic stress affects the brain and body (81%), and the relationship between mental health and trauma (86%). Fewer partners agreed that their partnership offered training on how to develop crisis and prevention plans (59%). Training practices were included early in the initiative; regression analysis showed that partnership members received on average .8 additional trainings per six month-period (*p* = .01, data not shown).

**Table 2:** Training in Trauma-Informed Practices among Partnership Members (*N* = 332)

Trauma-Informed Practice	Partnership Incorporates Practice, %
What traumatic stress is	85%
How traumatic stress affects the brain and body	81%
The relationship between mental health and trauma	86%
The relationship between substance use and trauma	69%
The relationship between homelessness and trauma	72%
How trauma affects a child’s development	80%
How trauma affects a child’s attachment to his/her caregivers	67%
The relationship between childhood trauma and adult re-victimization (e.g., domestic violence, sexual assault)	69%
Different cultural issues (different cultural practices, beliefs, rituals)	69%
Cultural differences in how people understand and respond to trauma	69%
How working with trauma survivors impacts all of us	75%
How to help community members identify triggers (e.g., reminders of dangerous or frightening things that have happened in the past)	68%
How to help community members manage their feelings (e.g., helplessness, rage, sadness, terror, etc.)	71%
De-escalation strategies (i.e., ways to help people to calm down before reaching the point of crisis)	69%
How to develop safety and crisis prevention plans	59%
How to establish and maintain healthy boundaries	72%
How to understand the relationship between poverty and trauma	69%
Understanding how poverty and trauma may impact cognitive abilities, behavior and/or engagement (in treatment, school, work etc.)	70%
How to develop a common vocabulary to describe the impact of stress and trauma on individuals	70%

Table 3 provides a summary of community capacity-building activities from January 2018 through October 2020. Capacity-building occurred both within the partnerships and within the community. Within the partnerships, capacity-building included partnership meetings and trainings in trauma-informed practice for partnership members. Partnership meetings were used to collaboratively develop programming, problem-solve, and share resources among partners. The nine partnerships averaged 76 meetings per month, with an average of 10 attendees per event. Trainings for partnership members focused on increasing knowledge about trauma and strategies to reduce the impact of trauma. Trainings included the Community Resiliency Model, Mental Health First Aid, Trauma-Informed Care, and Trauma Resilience [17, 26, 27]. Combined, the nine partnerships averaged 26 trainings per month and 16 attendees per event.

**Table 3:** Partnership and Community Capacity-Building Activities from January 2018 through October 2020

Type of Event	Number of Events	Events per Month, Mean (SE)	Number of Attendees	Attendees per Event, Mean (SE)
*Partnership Capacity-Building*				
Partnership Meeting	1741	76 (13)	18,226	10 (1)
Training for Partnership Members	650	26 (5)	9406	16 (2)
*Community Capacity-Building*				
Community Outreach	2190	91 (20)	175,763	80 (46)
Community Event	460	21 (3)	19,154	42 (5)
Group Activities in the Community	1545	70 (15)	16,495	11 (1)
Training in the Community	1248	45 (11)	14,969	12 (1)

Capacity-building within the community aimed to increase community awareness of the impact of trauma, and available resources and supports for those who have experienced trauma or significant adversity. Community capacity-building included community outreach, community events, group activities and trainings in the community. Community outreach was varied and included attendance at neighborhood meetings, meal delivery at motels and drop-in centers, mobile showers and laundry opportunities for homeless youth, and cultivating relationships with local businesses, administrators and educators at schools, community leaders and other community organizations to grow the partnership’s resource network. From January 2018 through October 2020, the nine partnerships conducted 2190 outreach activities with a total of nearly 176,000 attendees.

Community events were informal engagement opportunities for partnership members to interact with large groups of community members, “meet people where they are at,” share information, conduct wellness screenings and increase awareness of available resources and supports. Community events included family fun nights providing a dinner and music or family friendly arts and crafts, BBQs in the park, drive-up back to school events where children received backpacks with virtual learning supports such as headphones, drop-in events for youth, and mindfulness hangouts. Partnerships conducted 460 community events with a total of over 19,000 attendees.

Group activities included ongoing programming and classes which were designed to foster social connectedness and resilience. Group activities included parenting classes to increase knowledge of child development, play groups for children to support healthy attachments, knitting and storytelling circles for intergenerational families who have experienced trauma, yoga and Zumba classes to promote wellness, and skills trainings to support individuals who are seeking employment or education. After issuance of stay-at-home orders due to COVID-19, partnerships supported community members’ connections and participation in virtual activities using Zoom or social media platforms (Facebook Live, Instagram). Partnerships conducted 1545 group activities with a total of nearly 16,500 attendees.

Trainings in the community were meant to build awareness of trauma and its impact. Examples include anger management, addressing domestic violence, developing a trauma-informed curriculum in the public school system, mindfulness practices for educators and school staff, and strategies for identifying and engaging with someone who experiences a mental health crisis within the law enforcement or court system. During the pandemic, training expanded to include COVID-19, racial injustice and health disparities, the use of technology to connect virtually, and taking care of mental health during extreme times of stress. Partnerships conducted 1248 trainings with a total of nearly 15,000 attendees.

**Fig. 1:**
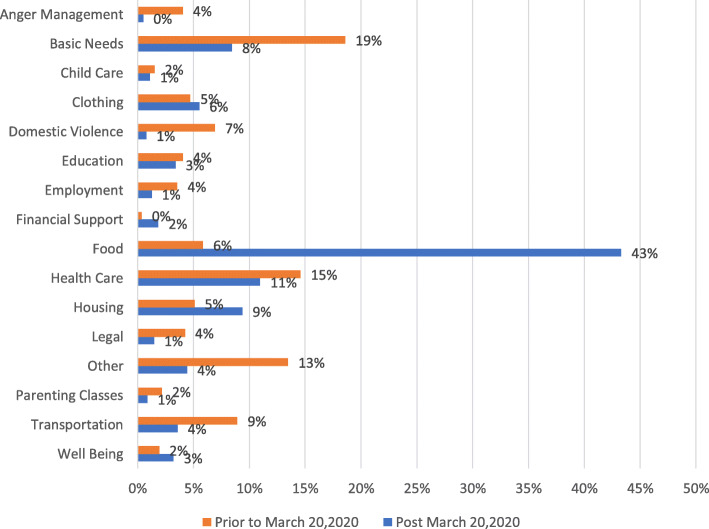
Distribution of 13,847 Linkages of Community Members to Resources and Services from January 2018 through October 2020

Figure 1 shows the distribution of nearly 14,000 linkages to services before and after stay-at-home orders were issued in California. Prior to March 20, 2020, the most common service linkages made were for basic needs (19%, e.g., personal hygiene or household products or diapers), and physical and mental health care (15%). There was a significant shift in the types of linkages after the stay-at-home orders were issued (*p* < .01); from March 20, 2020 onward, the most common linkage was for food distribution (43%). There were increased linkages for housing (from 5 to 9%) and decreased linkages for anger management training (from 4 to 0%) and support related to domestic violence (from 7 to 1%) (*p* < .01 each). Community members’ follow-through on linkages remained consistent pre-COVID-19 (85%) and during the pandemic (87%) (data not shown).

Table 4 shows changes in three stress tolerance self-reports assessing trauma-related resilience, coping skills, and social connectedness among 699 participants who provided two responses to each measure between 30 days and 180 days apart (mean = 99 days, SD = 33) using paired t-tests. We found no changes in resilience as measured by the unidimensional CD-RISC 10 score. On the Brief COPE, significant changes occurred only in external help-seeking: use of Emotional Support (*P* = .024) and Informational Support (*P* = .003). Social connectedness increased on ICS (*P* = .015).

**Table 4:** Measures of Trauma Resilience Among Community Members Engaged in the Partnerships (*N* = 699)

	Baseline		Follow-up		Difference	
Measure	Mean Score (SE)	Range	Mean Score (SE)	Range	Mean Score (SE)	*P*-Value
CDRISC-10	28.4 (.3)	0–40	28.6 (.3)	3–40	.2 (.3)	.476
BRIEF COPE:						
Emotional Support	5.5 (.07)	2–8	5.7 (.07)	2–8	.2 (.08)	.024
Informational Support	5.6 (.07)	2–8	5.8 (.07)	2–8	.2 (.08)	.003
Active Coping	6.3 (.06)	2–8	6.3 (.06)	2–8	−.04 (.07)	.586
Planning	6.0 (.07)	2–8	6.1 (.07)	2–8	.1 (.08)	.207
ICS	3.4 (.06)	1–6	3.6 (.06)	1–6	.2 (.06)	.015

## Discussion

These findings add to the growing literature on community-wide efforts to broaden the scope of trauma-informed practices beyond single agencies [19]. Specifically, we examined the initial lessons gleaned from an innovative effort by LACDMH to support community well-being through partnerships aiming to build capacity to address the impact of trauma within their communities. These partnerships are comprised of community-based organizations engaged in trauma-informed capacity-building strategies within their organizations that were specific to the needs of their communities. The primary goals of this initiative were empowerment of the community to take collective ownership and action in creating awareness of how trauma impacts the individual and the community and promoting the health and well-being of their community members. The capacity-building strategies pursued in service of this goal include building trauma resilient families; offering trauma-informed education and support for school communities; creating support networks for transitional age youth, justice involved individuals, and older adults; and providing culturally competent activities for multigenerational families experiencing trauma. These strategies do not, however, reflect direct delivery of mental health interventions.

A critical step in implementing community-wide projects with fidelity is to provide foundational education and training opportunities [28]. Trauma-informed practices support the engagement and education of community members through the application of the core principles of safety, trustworthiness and transparency, collaboration and mutuality, empowerment, choice, and cultural competence and humility [16, 29, 30]. We found that training and education opportunities in trauma-informed practices were widespread in the partnerships, with 66% of partnership members reporting training in 13 or more trauma-informed practices. Most partners endorsed having training opportunities to learn about traumatic stress and its impact on child development, and mental and physical health. The training opportunity with the least availability across the partnerships was the development of safety and crisis prevention plans (59%). In light of a global pandemic and national civil unrest, combined with the core trauma-informed principle of safety, this finding underscores the need to establish training that facilitates preparedness for large-scale community responses to disruption in service and access to basic necessities such as food and water. Further, safety planning skills are essential for frontline staff conducting outreach within the community.

Partnerships’ capacity-building activities focused on creating awareness about the impact of trauma both within the partnership and in the community, disseminating information about resources available within the community, and utilizing trauma-informed principles to develop trust so that partnerships are seen as reliable, accessible, and legitimate leaders in the community. Activities were tailored to each community and included trust building, collaborative, and choice-based opportunities. Examples included engaging community members to identify their own needs and most valued activities, empowering community members to lead group activities, and employing peers from the community to build trust and foster engagement of community members who may not otherwise seek mental health or other social services.

During the pandemic, partnerships pivoted to find new ways to build community and connect with community members. Providers would check-in weekly by phone or text message to maintain a connection with families and support emerging needs even though community activity groups no longer met in person. For the youth community, providers expanded their programming using Facebook and Instagram live for group activities that were created and facilitated by youth peers; new online group activities included yoga and nutrition, a cooking group, book club, a virtual gym, and a video gaming group. Since the onset of the pandemic, partnerships report that a wider range of community members now directly seek support through word of mouth and social media, likely because of the relationships and trust-building with the community prior to COVID-19.

Community members were consistently engaged in their follow-through on linkages and referrals. Successful linkages were made for a diverse set of resources reflecting the needs specific to each community both pre- (85%) and during (87%) COVID-19. Notably, more linkages were made for concrete supports in the seven months after the stay-at-home orders were issued in California than in the prior 20 months. Regardless of the primary capacity-building strategy initially embraced and implemented by each partnership, all partnerships pivoted to assisting community members with their basic needs, such as food and shelter, during COVID-19. The foundational capacity-building work of the community partnerships, along with the community social capital each partnership was establishing prior to the pandemic, allowed for a natural transition to COVID-19 related community support.

The trauma-informed nature of the linkages is three-fold. First, the trauma-informed training delivered to partnership members facilitated their ability to successfully identify community needs through trust, empowerment, and community collaboration. For example, partnership members listened to what the community members voiced as their most pressing needs, which changed during COVID-19, and responded collaboratively according to the community’s self-identified needs, supporting their voice and choice. Second, trauma-informed training to partnerships and the communities provided a common language for thinking about and addressing trauma within the partnerships and between partnership members and the community. A common language and shared understanding about trauma’s impact are likely to increase buy-in and engagement, and to result in more successful linkages. Third, the linkages directly reflect adversity-driven needs (employment, food, housing), that are often associated with large-scale societal issues (poverty and discrimination) and amplified by a wide range of other traumatic events (domestic violence, child maltreatment).

The technology-based pivot in outreach strategies, combined with increased linkages in response to COVID-19, demonstrate the underlying strength, innovation, and responsiveness of the capacity-building approach. Partnerships had already laid the groundwork and established trust within their communities, resulting in a nimble, local response to a global crisis. Some agencies added new partners and formed new relationships with community businesses to sustain linkages with needed resources as the pandemic persisted. The partnerships’ response to the pandemic shows how organizations that are part of a network are able to leverage resources, new ideas and knowledge to respond to community needs more effectively than those that “go it alone” [18]. For example, as stay-at-home orders were implemented and homeless shelters began to close due to COVID-19, one partnership reached out to a local motel to temporarily house transition aged youth and older adults. This same partnership worked with the local school district to provide daily meals to community members at the community center. This level of support likely would not have been sustainable if actioned by a single organization.

In contrast, linkages declined for anger management training and support related to domestic violence. In-person resources for problems like intimate partner violence were extremely limited across the US during the pandemic [31]. While sensitive trainings pose notable challenges in a virtual-forum delivery format, current data indicate increased intimate partner violence during periods of social crisis and uncertainty, as public health measures during the pandemic may quarantine victims with their abusers, and even prevent technology-based help-seeking by phone or computer [32, 33]. Capacity-building efforts should include contingency planning with community members as well as exploration of innovative ways to safely check-in on families when traditional resources are stretched thin.

In terms of stress tolerance capacities, we found that trauma-related resiliency was not diminished over time even though most follow-up measures (88%) occurred during a global pandemic. These data align with trauma-informed community resilience models and collaboratives that find communities are imbued with numerous facets of resiliency that can be built upon and broadened through the sharing of existing skills among community members [34]. In terms of coping skills, small but significant gains occurred on external indicators of help-seeking, specifically the increased use of emotional and informational supports to cope with stress. This aligns with the trust building aspects of the partnerships’ community engagement practices and conceptualizing resource linkages as fostering help-seeking behaviors [35].

In contrast, no changes occurred on coping strategies that reflect an internal locus of control, such as active coping (“*I’ve been taking action to try to make the situation better*”) and planning (“*I’ve been trying to come up with a strategy about what to do*”). While the Brief COPE does not query the specific situation that respondents have in mind, it may reflect that community-wide, stressful events such as COVID-19, civil unrest and changes in government leadership fall outside of any individual’s direct control. Further, the partnerships’ primary community capacity-building strategies embrace engagement, linkages, education, and very specific population needs, rather than provide direct delivery of mental health services or coping-specific interventions.

Consistent with reported increases in seeking emotional support to improve coping, small improvements were also observed in perception of community connectedness, despite pandemic-related physical distancing. Overall, our findings indicate no loss of stress tolerance, and even some gains, during these stressful times. Combined with increased linkages and sustained linkage success, improvements in social connectedness and help-seeking stressor tolerance indicators hold promise for how community capacity-building efforts can prepare members for unanticipated large-scale stressors, even when strategies may vary by community. Folding in specific stress tolerance strategies, taught with explicit intentionality, and providing scaffolding for community members about the use of different coping techniques, could further enhance individual-level skills when implementing broader community capacity-building efforts.

There are inherent limitations to this evaluation. The participating partnerships, and their community impact, are context specific. The efforts of one partnership among a specific group in a local area may not translate to other partnerships, community groups, or local areas. Even though data were obtained from one county, Los Angeles County is geographically large (~ 4000 mile^2^), the most populous county in the US, larger in population than 42 states in the US and encompasses 188 cities and unincorporated communities that range from rural to densely urban. We were not able to randomly assign community members to the initiative. As such, it is challenging to identify a community-level effect for the initiative, and to isolate that effect from significant changes within in the community, such as those driven by COVID-19.

The initiative leveraged community providers. Yet, the quantitative data provided in this paper do not explain how training in trauma-informed practices impacted providers’ perceptions of community members. Health and human service agencies have often been traumatizing and discriminatory in their service delivery. Although the TIOT includes measures of cultural competency, it would be useful to know how changes in community providers’ perceptions of community members affected community outcomes. This question will be explored in future papers using qualitative data collected as part of the larger evaluation. We did not have sufficient statistical power to test for differences in impact among the capacity-building efforts using quantitative data alone; however, we intend to pursue this line of inquiry using the qualitative data generated under the larger study.

## Conclusions

We found that capacity-building among community-based partnerships is effective at disseminating trauma-informed education and training, conducting outreach and engagement, linking community members with resources, and increasing help-seeking and social connectedness by community members. Some key questions remain, including whether community capacity-building is an effective and sustainable strategy for addressing the consequences of trauma or for supporting mental health and wellness within communities. As we look to the future, can the community capacity-building and specific strategies help communities mitigate the negative emotional impacts of inevitable future natural, man-made and/or biological events that result in trauma reactions? In other words, is community capacity-building a foundational competency that can mitigate the impact of natural disasters such as earthquakes, fires and flooding or future acts of social injustice? Our qualitative work will build on the present findings and examine the impact and sustainability of this community capacity-building approach to addressing trauma within communities. Future studies should examine the relationships among community stress tolerance capacities and community capacity-building strategies designed to develop safe spaces, build trust, and normalize help-seeking skills.

## Data Availability

Access to the datasets generated and analyzed during the current study are restricted to protect the confidentiality of the participants. These datasets can be made available by the authors conditional on approval by LACDMH.

## Abbreviations

LACDMH: Los Angeles County Department of Mental Health
TIOT: Trauma-Informed Organizational Toolkit
CD-RISC 10: Conner Davidson Resilience Scale
Brief COPE: Coping Orientation to Problems
ICS: Inclusion of Community in Self Scale
